# Prediction of split renal function and obstruction with magnetic resonance urography in comparison with dynamic renal scintigraphy

**DOI:** 10.1007/s00467-026-07211-y

**Published:** 2026-02-17

**Authors:** Seckin Cobanoglu, Hazal Karli, Emine Goknur Isik, Eda Cingoz, Ravza Yilmaz, Zuhal Bayramoglu

**Affiliations:** 1 Radiology Department, Manavgat State Hospital, Antalya, Turkey; 2 Radiology Department, Istanbul Bagcilar Training and Research Hospital, Istanbul, Turkey; 3 Istanbul Medical Faculty, Nuclear Medicine Department, Istanbul University, Istanbul, Turkey; 4 Istanbul Faculty of Medicine, Radiology Department, Istanbul University, Istanbul, Turkey

**Keywords:** MR urography, MAG3 scintigraphy, Renal function, Children

## Abstract

**Background:**

Magnetic resonance urography (MRU) examinations allow the assessment of kidney functions through recently developed image post-processing techniques, in addition to providing detailed visualization of urinary system anatomy. MRU and MAG3 scintigraphy were compared in terms of functional evaluation.

**Methods:**

Dynamic contrast-enhanced MRU images of 76 children who had previously undergone MAG3 scintigraphy examinations were evaluated. Morphological parameters, volumetric split renal functions (SRF), and renal transit times (RTT) were calculated from the dynamic MRU phases using the CHOP-fMRU software. Results were compared with the half-time (T1/2) and SRF values obtained from MAG3 scintigraphy. Student’s t-test and Mann–Whitney U test were used to compare quantitative variables, and Pearson correlation analysis was performed. ROC analysis was performed to reveal the prediction of obstruction by MRU-based quantitative parameters.

**Results:**

The ages of included patients ranged from 1 to 216 months (median: 59.50 months, IQR = 4.1–179.5 months). Statistically significant positive correlations (r > 0.9, p = 0.001) were found between SRFs of the right and left kidneys, measured by MRU and scintigraphy. When the cut-off value was set to 11.5 min for RTT, the sensitivity, specificity, positive and negative predictive values, and accuracy of RTT were found to be 94%, 86%, 90%, 88%, and 88%, respectively.

**Conclusion:**

MRU could be an invaluable tool in the assessment of renal functions. Its diagnostic accuracy in detecting the level and extent of obstruction is comparable to that of MAG3 scintigraphy. Despite the lack of extensive comparative data, the potential benefits of MRU justify the need for further studies.

**Graphical abstract:**

**Figure:**
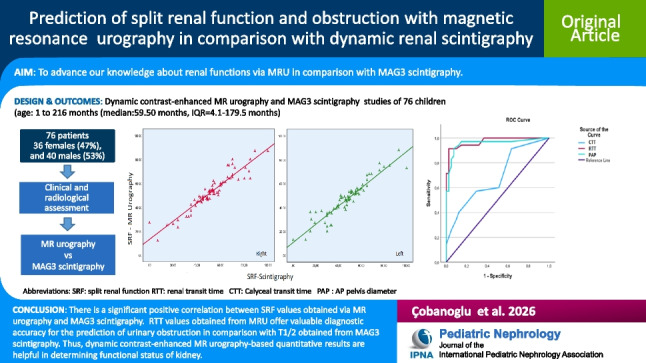
A higher-resolution version of the Graphical abstract is available as  Supplementary information

**Supplementary Information:**

The online version contains supplementary material available at 10.1007/s00467-026-07211-y.

## Introduction

Magnetic resonance urography (MRU) is extremely important for detecting congenital malformations and acquired pathologies related to the kidneys and urinary tract in pediatric patients. Its non-invasive nature and lack of ionizing radiation make it a safe option, while its ability to provide detailed morphological and functional assessments facilitates comprehensive care [1, 2]. Congenital malformations present a wide range of challenges, from harmless renal ectopia to potentially fatal bilateral renal agenesis. Rapid diagnosis and functional evaluation are crucial to determine appropriate treatment and achieve early functional recovery. This is particularly important because concomitant obstructive uropathy is a leading cause of renal failure in infancy [3]. By prioritizing early intervention, we can significantly improve outcomes and protect the health of our youngest patients.

Ultrasonography (USG) and computed tomography (CT) are the primary radiological techniques used to assess the structure of the kidneys and urinary tract. However, for a more in-depth understanding of renal function, MRU stands out by providing critical functional data. Additionally, the voiding cystourethrogram (VCUG) not only offers vital anatomical details but also enables dynamic evaluation of the urinary system. Adopting these methods ensures a comprehensive assessment for optimal patient care. Integrating radionuclide scintigraphy with radiologic techniques in the evaluation of renal function significantly improves diagnostic accuracy. Notably, dimercaptosuccinic acid (Tc-99 m-DMSA), mercaptoacetyltriglycine (Tc-99 m-MAG3), and diethylenetriaminepentaacetic acid (Tc-99 m-DTPA) are vital tools in nuclear medicine, providing invaluable insights into renal health [4]. USG is a widely used tool in the evaluation of the urinary system in children and performs effectively in identifying mildly and moderately dilated collecting systems. However, it falls short in evaluating duplicated or malrotated collecting systems or severely dilated collecting systems, highlighting the need for alternative diagnostic options to ensure comprehensive care and accurate assessment. It is not feasible to assess split renal function (SRF) using USG because it is not possible to determine the percentage of non-functional renal tissue and volumetrically evaluate the affected kidney using USG. MAG3 dynamic renal scintigraphy is considered the definitive gold standard for accurately assessing SRF and effectively identifying obstructions, making it an indispensable tool in modern renal diagnostics [5]. Although MAG3 scintigraphy can effectively assess SRF and the collecting system, radiation exposure and poor anatomical detail due to low spatial resolution are limiting factors for its use.

MRU, due to its high spatial and contrast resolution, is an effective imaging technique for evaluating urinary system anatomy. The presence of an obstruction can be revealed through the anatomical details it provides, along with the location of the obstruction. MRU also provides functional data through post-processing image analysis [6]. MRU is increasingly utilized, due to rapid imaging sequences that enable image acquisition during breath-hold periods and contrast medium–enhanced studies that include dynamic measurements [7]. MRU is a beneficial imaging modality for the urinary tract in children, providing comprehensive anatomical and functional data while producing relatively high-quality images without the use of ionizing radiation. On the other hand, a MAG3 scan implies a very low radiation burden, far below 1 mSv, a harmless amount according to the International Commission on Radiological Protection recommendations. The lifetime radiation risk associated with a 99mTc-MAG3 scan has been found to be very low in comparison with the general population’s risk for developing cancer [8].

The literature reveals a remarkable paucity of comparative and quantitative functional studies that directly assess MAG3 scintigraphy versus contrast-enhanced MRU. This lack of research contributes to a limited understanding of how MRU performs compared to MAG3 scintigraphy and highlights the need for further investigation in this area [9–11]. This study aims to significantly advance our knowledge of SRF by comparing MRU and MAG3 scintigraphy. Moreover, it aims to provide valuable insights into obstruction prediction.

## Materials and methods

In the current study, we evaluated dynamic MRU and dynamic renal scintigraphy using MAG3 in 76 patients. These patients were followed for congenital urinary system anomalies at our hospital between 2006 and 2021. None of the patients had a history of urinary tract infection or surgical intervention within the previous six months. The evaluation was conducted retrospectively by a nuclear medicine specialist and two radiologists, one of whom is board-certified. Informed consent was waived due to the retrospective nature of the study. The study was approved by the local Ethics Committee of Istanbul Medical Faculty on October 12, 2021 (File Number: 22).

MRU examinations were performed in the supine position using a 1.5 Tesla device (Magnetom Symphony and Magnetom Aera, Siemens Healthcare). A head coil or a phased array receiver coil was used, depending on the patient's body size. After localizer images were obtained in all three planes, coronal and axial plane images were obtained using a HASTE (single-shot fast spin echo) sequence. Imaging began at the superior aspect of the diaphragm and continued down to the bladder. T1 and T2-weighted images were obtained, and the static examination was completed with heavy T2 sequences in the coronal plane. A T1-weighted 3D gradient echo sequence was used for dynamic analysis. Our clinic’s hydration protocol for MRU examination includes the administration of 20 mL/kg of saline 30 min before the study, followed by 10 mL/kg during the examination. After ensuring that patients were well-hydrated and had satisfactory urine output, intravenous furosemide was administered at a dose of 1 mg/kg to facilitate visualization of the collecting system. Following initial images obtained without contrast material, a contrast agent (gadobutrol and gadoterate meglumine) was administered as an IV bolus at a dose of 0.1 mmol/kg. Dynamic examinations were completed by obtaining additional images.

MRU scans were scheduled around the bedtime of the infants who had just been fed and swaddled according to the “feed and wrap technique” [12]. The presence of urine in the bladder or diaper supports adequate hydration in infants. Since the density of contrast material is higher than that of water, it is likely to create a dependent level, especially in the presence of stasis and the accumulation of the urine in the pelvis due to obstruction or high-grade reflux. Total acquisition time to visualize contrast excretion from the UPJ varies because of the signal drop due to contrast hyperconcentration, especially in severely dilated systems. The MRU scans were performed within 30–45 min following contrast administration and may take up to 60 min in the presence of prolonged excretion due to advanced dilatation. Since anesthesia is required for children unable to adapt to the procedures, particularly under 5 years of age, the recording was terminated after 45 min to avoid the risk of hypoventilation.

The diuretic renal scintigraphy utilized Tc99m-MAG3, which is the preferred radioisotope for the pediatric population. Before the procedure, patients received oral or intravenous hydration, depending on their age. Posterior dynamic images were captured after injecting 3.7 MBq/kg of the radionuclide agent and 1 mg/kg of furosemide (with a maximum dose of 20 mg). A comprehensive evaluation of urine drainage was performed in a post-void image of a scintigraphy. A nuclear medicine specialist evaluated the images and determined Tmax, T1/2, and SRF values.

Urinary tract morphology was assessed using various parameters during MRU examinations, including parenchyma thickness, anterior–posterior diameter of the renal pelvis (APRPD), and kidney dimensions obtained from conventional sequences. Temporal and quantitative data regarding SRF, calyceal (CTT), and renal transit time (RTT) were gathered using the semi-automated MRU software developed by the Children's Hospital of Philadelphia (CHOP). SRFs obtained by MRU were compared with the results of MAG3 scintigraphy. To assess urinary system obstruction in the same patient group, we evaluated T1/2, which represents the time required for renal activity to decrease to 50% of its maximum value, calculated by MAG3 scintigraphy, and RTT, which represents the time required for the contrast agent to be visualized in the ureteral segment below the lower pole of the related kidney, observed in the arterial phase of the renal cortex on dynamic post-contrast imaging [13]. Considering the RTT, urinary tract obstruction detection was classified as normal (RTT ≤ 245 s), suspicious (245 < RTT ≤ 490 s), or obstructed (RTT > 490 s) as given in the latest study [14]. In scintigraphy, which is the reference method for demonstrating obstruction, cases with a T1/2 time greater than 20 min suggesting upper urinary tract obstruction were selected, and the sensitivity, specificity, and positive and negative predictive values in the assessment of urinary tract obstruction were analyzed.

Statistical analyses were performed using the NCSS (Number Cruncher Statistical System) program. Study data were evaluated using descriptive statistical methods (mean, standard deviation, median, minimum, and maximum). The Shapiro–Wilk test and graphical analyses were used to ensure compliance of quantitative data with normal distribution. The Student t test was used to compare quantitative variables with normal distribution between two groups, and the Mann–Whitney U test was used to compare quantitative variables with non-normal distribution. The McNemar goodness-of-fit test and diagnostic screening tests were used to compare qualitative data. Pearson correlation analysis was used to evaluate relationships between quantitative variables. Bland–Altman plot was used to evaluate the agreement between the values obtained by different imaging techniques. ROC analysis was plotted for determination of obstruction via MRU-based quantitative parameters when MAG3 scintigraphy is taken as a reference method. Statistical significance was accepted as p < 0.05.

## Results

A total of 76 patients, 36 females (47%), and 40 males (53%), participated in the study. The ages of the patients included in the study ranged from 1 to 216 months (median: 59.50 months, IQR = 4.1–179.5 months). Thirty patients were younger than 3 years old, with a mean age of 14.9 months (range of 1–36 months).

The diagnoses made to the patients as a result of clinical evaluation and MR imaging are given in Table 1. The morphological evaluation results regarding the parenchymal thickness, renal pelvis, and ureter diameter in each ureteropelvic unit are given in Table 2.

**Table 1:** Diagnosis of the kidneys and number of ureteropelvic units included in the study

Diagnosis	Cases (n)	Percent (%)	Number of ureteropelvic units
Ureteropelvic junction obstruction	37	48.6	74
Ureterovesical junction obstruction	6	7.89	12
Ureterocele	2	2.6	4
Double collecting system	10	13	30
Double collecting system + ureteropelvic junction obstruction	5	6.6	15
Vesicoureteral reflux	7	9.2	14
Congenital megaureter	4	5.3	8
Horseshoe kidney	2	2.63	4
Posterior urethral valve	1	1.3	2
Renal hypoplasia	1	1.3	2
Bladder extrophy	1	1.3	2
Total	**76**	**100**	**167**

**Table 2:** Evaluation of morphological parameters in ureteropelvic units

		Left (n:82)	Right (n:85)	p
Parenchymal thickness (mm)	Mean ± SD	9.86 ± 9.4	10.2 ± 9.12	^*b*^*0.3*
	Median (Min–Max)	8.4 (0–87)	9.7 (0–85)	
AP diameter of renal pelvis (mm)	Mean ± SD	13.05 ± 11.54	11.98 ± 8.25	^*b*^*0.4*
	Median (Min–Max)	7.8 (3–5.2)	8.7 (2–51.8)	
Ureter diameter (mm)	Mean ± SD	5.73 ± 5.2	4.32 ± 4.56	^*b*^0.012*
	Median (Min–Max)	3.4 (0–28.8)	2.7 (1–23.7)	

^*a*^*Student t-Test, *^*b*^Mann–Whitney U Test, *p < *0.05 was depicted as statistically significant*

**Fig. 1:**
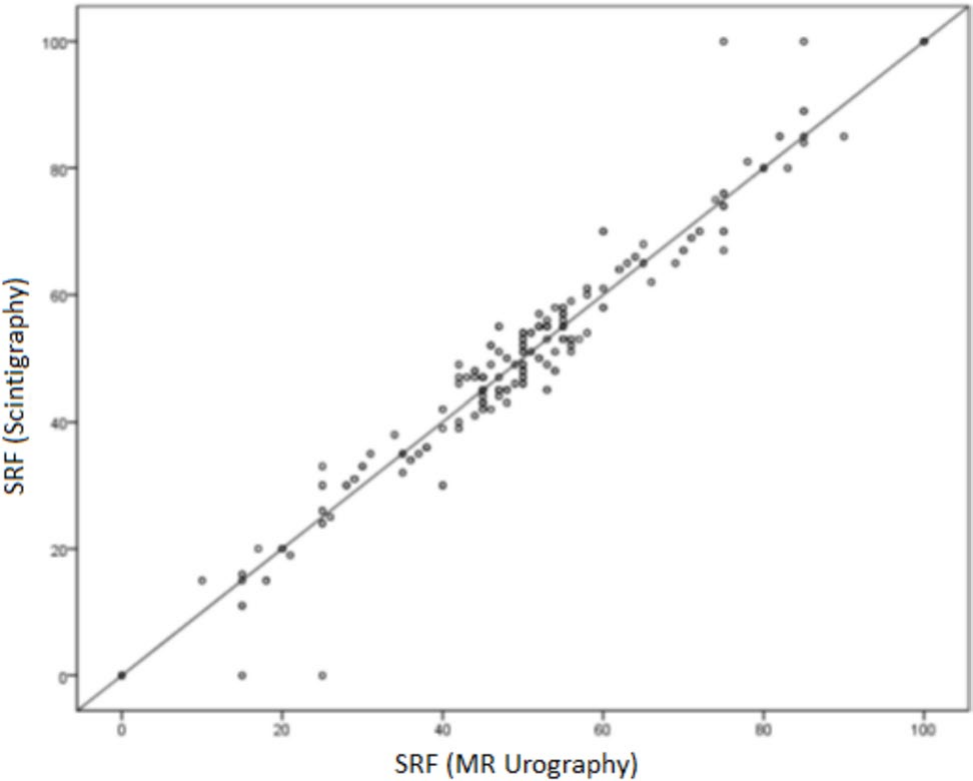
Distribution of the relationship between SRF measurements obtained by scintigraphy and MRU. Very strong positive correlations were found between the SRF measurements obtained by dynamic renal scintigraphy and dynamic MR urography (r = 0.97; *p* = 0.001)

**Fig. 2:**
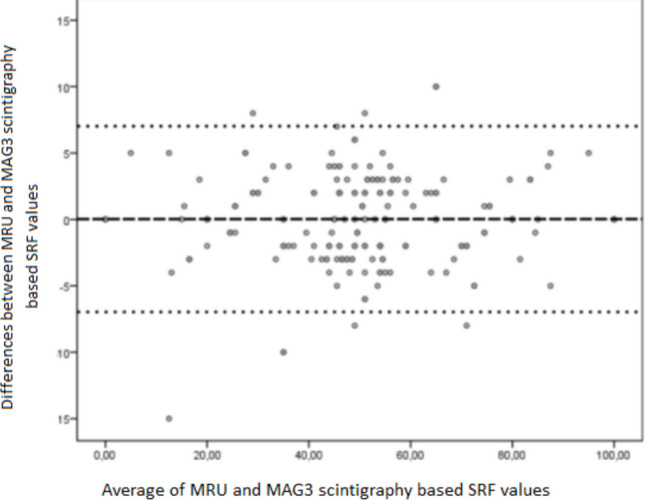
Bland–Altman plots analysis of agreement between MRU-based SRF and MAG3 scintigraphy-based SFR

A total of 76 right and 76 left kidneys were quantitatively evaluated on dynamic images. The mean time interval between scintigraphy and MRU examinations was 41 ± 29 days. The mean SRF measurements obtained by scintigraphy for the individuals participating in the study were 49.7 ± 19.7%. The mean SRF measurements obtained with functional MRU were 48.97 ± 18.75%. Very strong positive correlations were obtained between dynamic renal scintigraphy and dynamic MRU-based SRF values in general (r = 0.94; p = 0.001), in infants within the first 6 months (n = 15, r = 0.96; p = 0.001) and in children within 3 years (n = 41, r = 0.97; p = 0.001) (Table 3, Fig. 1). Bland–Altman plot analysis of interobserver reproducibility for SFR measurement shows strong agreement between the modalities (p = 0.56) (Fig. 2).

**Table 3:** SRF Measurements via dynamic renal scintigraphy and dynamic MRU

All cases	**SRF Scintigraphy**	Mean ± SD	49.7 ± 19.7	**p** 0.001	r* 0.93
		Median (Min–Max)	50 (10–90)		
	**SRF MRU**	Mean ± SD	48.97 ± 18.75		
		Median (Min–Max)	50 (12–88)		

^*^r = Pearson correlation coefficient

**Fig. 3:**
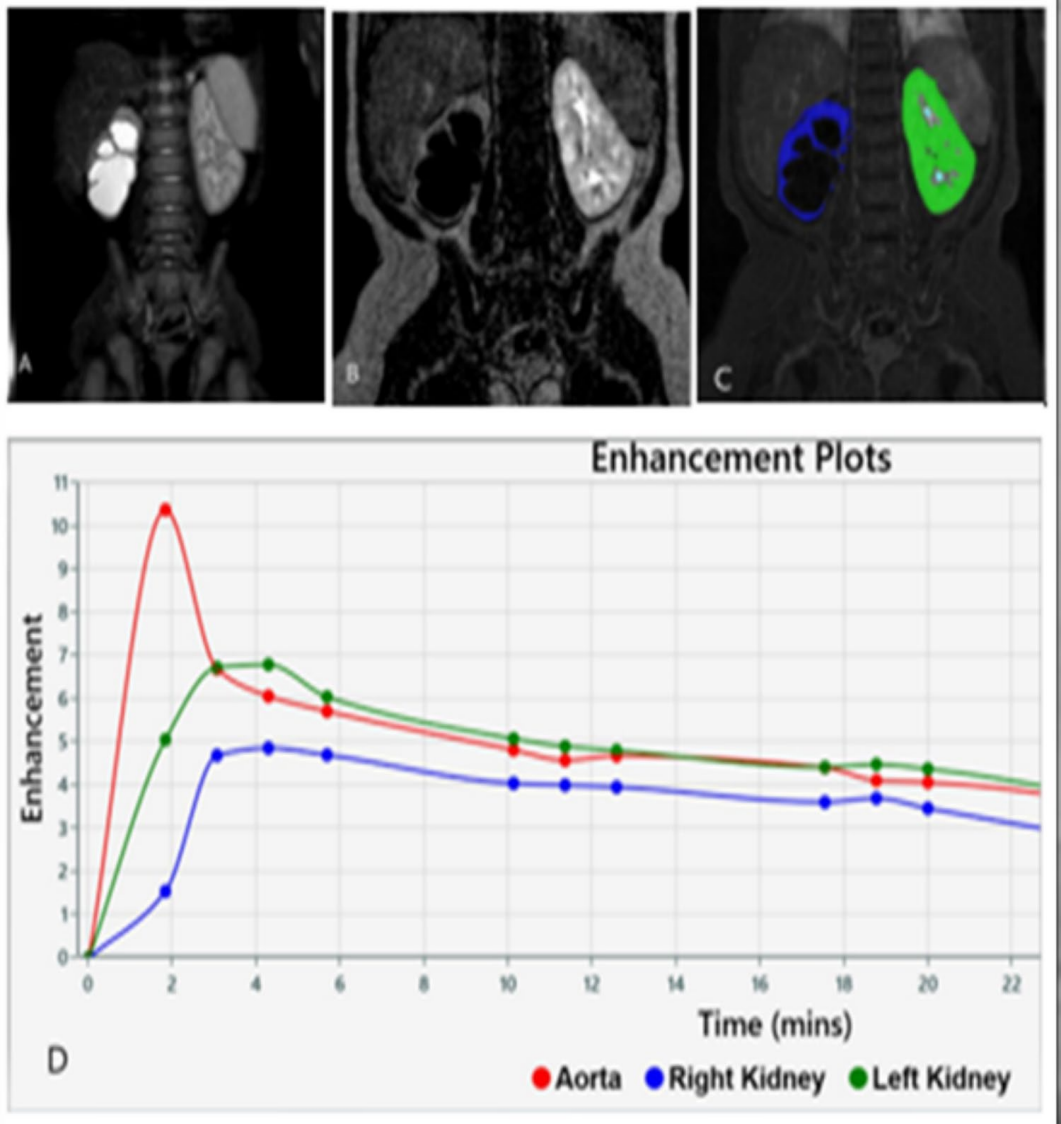
Functional MR urography imaging of a 9-month-old female patient followed for ureteropelvic junction obstruction. A–B. Dilated renal pelvis and calyx are observed in fat-suppressed coronal T2W and coronal dynamic T1W excretory phase images (although not visible in the images, proximal ureteral collapse). C. Automatic functional analysis of MR urography data after segmentation of the abdominal aorta, right (blue) and left (green) kidney parenchyma using CHOP fMRU software. D. Calculation of contrast curves. RTT values for right and left kidneys were 30 s vs. 2 s, respectively. Volumes of right and left kidneys were 21 mL and 53 mL, respectively. SRF values of right and left kidneys were 28.48% and 71.52%, respectively

Among all pelvicalyceal units, AP pelvis diameter presented highly significant good positive correlations with RTT (p = 0.001, r = 0.71), and moderate positive correlations with T1/2 (p = 0.001, r = 0.5), Tmax (p = 0.001, r = 0.55), and CTT (p = 0.001, r = 0.4). RTT presented highly significant moderate positive correlations with Tmax (p = 0.001, r = 0.54) and CTT (p = 0.001, r = 0.62), and significant moderate positive correlations with T1/2 (p = 0.02, r = 0.3). T1/2 presented highly significant good positive correlations with Tmax (p = 0.001, r = 0.84), and significant mild positive correlations with RTT (p = 0.01, r = 0.32) and CTT (p = 0.01, r = 0.34) (Fig. 3).

**Fig. 4:**
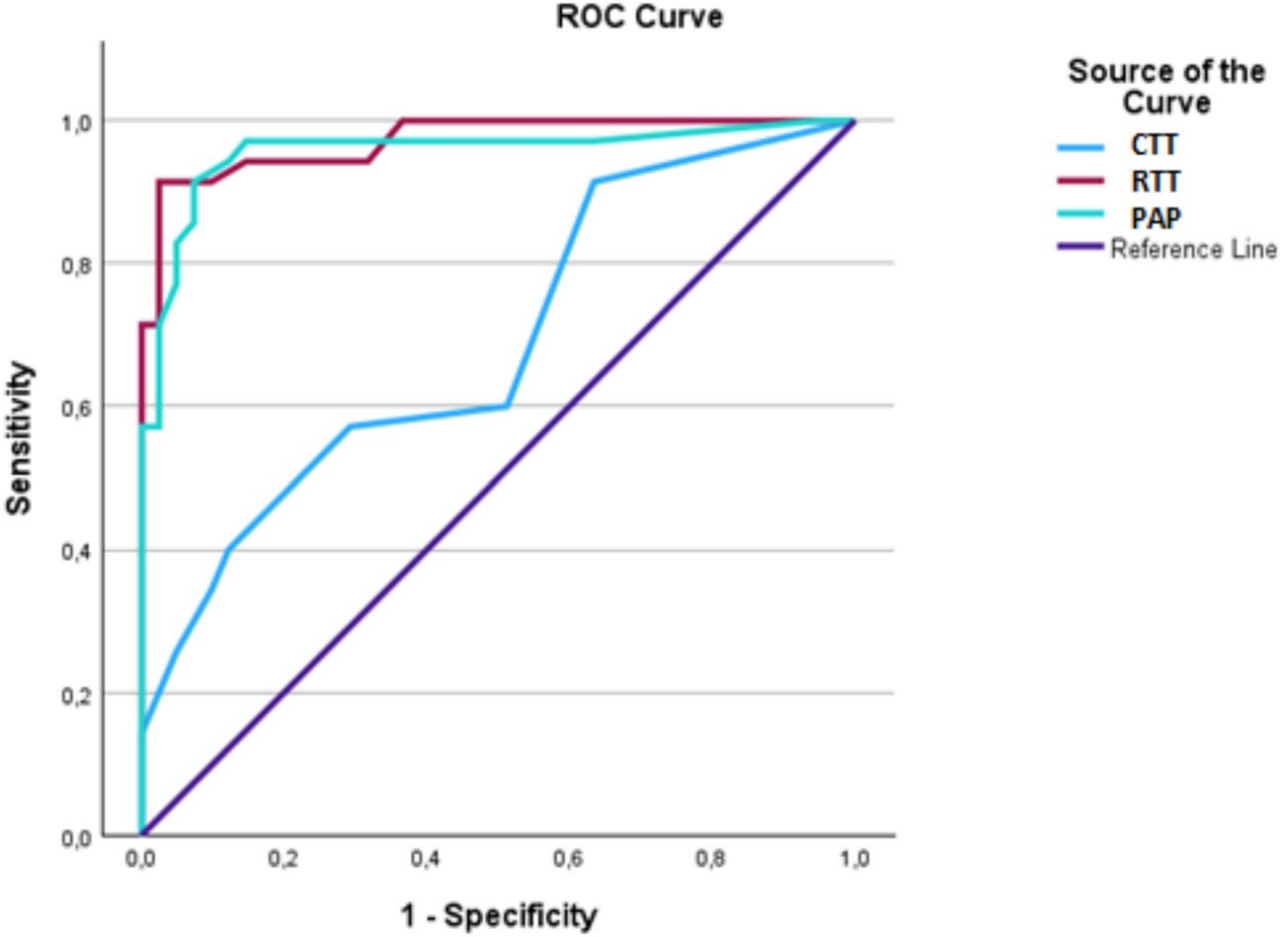
ROC analysis reveals diagnostic accuracy of CTT, RTT, and AP pelvis diameter in evaluation of urinary obstruction. CTT: Calyceal transit time, RTT: Renal transit time, PAP: AP pelvis diameter

Among all pelvicalyceal units, ROC analysis revealed significant diagnostic accuracies for RTT and pelvis AP diameter. Accordingly, when the cut-off value was set as 11.5 min for RTT; the sensitivity, specificity, positive and negative predictive values, and accuracy of RTT were found to be 94%, 86%, 90%, 88%, and 88%, respectively (Table 4). When the cut-off value was set as 14.5 mm for pelvis AP diameter; the sensitivity, specificity, positive and negative predictive values, and accuracy were found to be 91%, 82%, 89%, 92%, and 86%, respectively (Table 4, Fig. 4).

**Table 4:** Diagnostic accuracy of MRU-based quantitative values in the detection of obstruction

	Cut-off value	Sensitivity (%)	Specificity (%)	PPV (%)	NPV (%)	Diagnostic accuracy (%)	AUC (%)
CTT	4.5 min	71	70	68	65	68	69
RTT	11.5 min	94	86	90	88	88	97
AP diameter of renal pelvis	14.5 mm	91	82	89	92	86	94

Mc Nemar Test *p* < *0.01*

## Discussion

In our study, volumetric SRF was calculated by converting the enhanced renal parenchymal volume into percentages, with the assumption that the right and left kidneys account for 100% of the renal function via the software, and compared with MAG3 scintigraphy results, currently considered the reference method for renal function. SRF obtained from MRU examinations of the right and left kidneys showed highly significant positive correlations with MAG3 scintigraphy results. In addition, we achieved acceptable and promising diagnostic accuracy of MRU in the evaluation of urinary obstruction.

SRF represents the function of a kidney relative to total kidney function and is an important measure in renal health assessment. Renal scintigraphy is the standard method for determining SRF, and MAG3 scintigraphy is currently considered the gold standard due to its accuracy in detecting SRF [15]. This method not only detects SRF but also evaluates urinary tract obstructions. When used in conjunction with USG, which provides detailed anatomical information about the kidneys and collecting system, these techniques provide essential complementary information in pediatric kidneys [16]. However, it is important to consider the drawbacks of this combination, such as exposure to ionizing radiation during scintigraphy and the operator-dependent nature of USG, especially when it struggles to visualize both non-dilated and extremely dilated collecting systems. This limitation highlights the urgent need for alternative imaging modalities that enhance diagnostic accuracy and patient safety. There are several concerns on the long-term effect of ionizing radiation during scintigraphy. In renal scintigraphic examinations, the effective radiation dose varies between 0.7 mSv and 1.5 mSv, depending on age and body mass [17]. In comparison with adults, the pediatric patient data show a slightly higher radiation-related risk (excess lifetime risk) for the same absorbed doses. Overall, however, the lifetime radiation risk associated with the ^99m^Tc-MAG3 scans is very low when compared to the general population’s risk for developing cancer [18].

Recently, computed tomography urography (CTU) and functional MRU have been proposed as alternatives to conventional methods for estimating SRF in infants and children [14]. CTU-based estimation of SRF relies on the amount of contrast medium excreted by the kidneys. Shi et al. demonstrated that combining CTU images of the nephrographic phase with serum creatinine levels successfully determined SRF in adults [19]. You et al. revealed a good correlation between scintigraphy and CTU in terms of the determination of SRF, enabling the evaluation of renal function without additional radiation dose [20]. A comparison of CTU and routine reference scintigraphy to estimate SRF was evaluated in a prospective study conducted by Yuan et al. The findings were satisfactory and resulted in accurate measurements [21]. Nonetheless, CTU employs ionizing radiation, and there are contraindications to the use of iodine-based contrast medium in certain patient groups, necessitating the consideration of alternative modalities.

Recent studies have proposed the use of functional MRU as an alternative to conventional studies for evaluating SRF in patients with obstructive uropathy [9, 10, 16, 22]. Functional MRU is rapidly becoming a vital tool in daily medical practice, as it allows for the precise determination of morphological details. Its advanced capability to distinguish the signal intensities of soft tissue components provides exceptional soft tissue contrast, making it an invaluable asset for accurate diagnosis and treatment planning [23, 24]. This approach is highly effective in the management of complex congenital anomalies of the kidney and urinary tract (CAKUT) and offers the significant advantage of eliminating exposure to ionizing radiation [5]. Studies comparing scintigraphic methods and MRU in evaluating SRF have demonstrated a significant correlation between the two approaches. Furthermore, MRU stands out by providing precise measurements of the contrast-enhanced renal parenchymal volume, making it a valuable tool in clinical practice [11, 25]. Although MRU has a significant role in the assessment of SRF, due to the lack of comprehensive studies comparing the two techniques in the current literature, scintigraphic studies are still considered the reference method for the evaluation of obstructive uropathy as well as for guiding treatment decisions [5]. Damasio et al. found no significant difference in the drainage curves between MRU and scintigraphic evaluations in patients with CAKUT in children [5]. Rodigas et al. demonstrated that functional MRU offers significant diagnostic advantages, making it an essential complementary examination for challenging cases in infants and in children [16]. Claudon et al. demonstrated that MRU is as effective as scintigraphy for moderately dilated collecting systems [25]. Therefore, adopting MRU as a substitute for scintigraphy is not only advisable but also beneficial for enhancing diagnostic accuracy and efficiency in adults and children [25].

SRF measurements have been obtained via signal-intensity curves in dynamic contrast-enhanced MR nephrography, 3D gradient echo sequence with compressed-sensing and parallel imaging reconstruction by considering and grouping renal functions, non-contrast MRU derived texture parameters in comparison with scintigraphy, and integrated diffusion tensor imaging and renal parenchymal volume compared with scintigraphy [26–29]. Although some SRF results have been evaluated in comparison with scintigraphy, our study differs due to the patient group and methodologies, and direct comparisons cannot be made. In a study closely aligning with our findings, volumetric SRF calculated with CHOP-fMRU software demonstrated a significant correlation with MAG3 scintigraphy data in children. This research, which included 58 patients, mostly diagnosed with ureteropelvic junction (UPJ) obstruction (57%), highlights the reliability of these imaging techniques [30]. In a recent investigation by Jurkiewicz et al. on 46 pediatric patients, MRU-based SRF was compared with renal scintigraphy utilizing 99mTc-ethylenedicysteine (99mTc-EC). A strong agreement observed between these two methods further strengthens the reliability of our results and supports their use in clinical practice [31]. Additionally, a recent study comparing MRU with MAG3 scintigraphy to assess UPJ obstruction demonstrated impressive correlation coefficients regarding relative renal functions, highlighting the reliability of MRU as a diagnostic tool in children in this context [32].

Functional analysis data obtained from the two software programs, “CHOP-fMRU” and “Image J,” were meticulously compared with each other and with findings obtained from the 99mTc-Diethylenetriamine pentaacetate (DTPA) dynamic renal scintigraphy [33]. The results indicated that there were no statistically significant differences between CTT and RTT, renal parenchymal volumes, and volumetric SRF in children when CHOP-fMRU and Image J were compared (p > 0.05). Furthermore, the volumetric SRF values obtained from both CHOP-fMRU and Image J closely overlapped with the values obtained from the 99mTc-DTPA study, supporting the reliability of these imaging techniques. There is a distinct lack of quantitative and semiquantitative studies examining urinary obstruction using MRU in children. Utilizing parameters such as CTT, RTT, and mean transit time is crucial for a comprehensive quantitative assessment. Further research in this area could significantly improve our knowledge and management of urinary obstruction in pediatric patients. In a recent study investigating children, the RTT values in normal kidneys were found to range from 2.37 to 6.52 min in those with a ½ Tmax of less than 10 min as measured by MAG-3 scintigraphy. In contrast, kidneys exhibiting moderate uropathy, with a ½ Tmax value between 10 and 15 min, had a RTT range of 4.13 to 12.32 min in children [34]. No single definitive cut-off accuracy value has been established for ⁹⁹ᵐTc-MAG3 renal scintigraphy in infants aged 1–3 months. Diagnostic performance varies depending on the parameter being evaluated. While sensitivity to detect drainage disorders or obstruction is generally high (around 80–90%), specificity is lower in this age group (around 60–75%), due to the physiological immaturity of renal function. Differential renal function measurements, however, are considered more reproducible when studies are conducted under optimal conditions. In a study comparing UPJ obstruction using MRU and MAG3 scintigraphy, MAG3 scintigraphy was considered the gold standard for obstruction when a half-life (T1/2) of 20 min or greater was established. A study using a cut-off value of 6 min for RTT on MRU revealed a sensitivity of 61.9%, a specificity of 94.1%, and an impressive area under the curve value of 0.8271 [35]. These results highlight the effectiveness of MRU in evaluating UPJ stenosis in children alongside MAG3 scintigraphy [35]. In our study, when distal obstructions accompanied by ureteral dilation in addition to UPJ stenosis were included, we achieved high diagnostic efficacy for cases with RTT times of 11.5 min and above. In 8 patients diagnosed with ureterovesical junction stenosis due to distal ureteral dilation, an obstructive urogram pattern was obtained in 6 patients using both MAG3 and MRU data; in 1 patient using MRU data; and in 3 patients using MAG3 scintigraphy. Therefore, although these tests are quite distinctive in both diagnosing and ruling out obstruction, it should be considered that obstructive patterns can also occur in pathologies other than UPJ stenosis, as distal obstruction can significantly contribute to stasis.

In the current study, we represented acceptable and good correlations between MRU-based and MAG3-scintigraphy-based quantitative data. The remarkable correlation between the RTT values and T1/2 underscores its reliability, resulting in an impressive sensitivity of 100% and a specificity of 81.6%, making it a valuable tool in the diagnosis of urinary tract disorders in children [16]. Jones et al. reported that the RTT obtained from MRU examination and the half-life of renal signal decay obtained from dynamic renal scintigraphy were equally effective in predicting obstruction in children [14].

A T1/2 of less than 10 min can reliably exclude upper urinary tract obstruction [36]. However, a prolonged T1/2 interval cannot be considered a sufficient indicator for diagnosing obstruction on its own. T1/2 should be evaluated alongside the curves, postvoid images, urine flow rate, and other quantitative indices. A flat or rising drainage curve without clearance in the upright position could represent critical obstruction via scintigraphy [36]. In the current study, the drainage curves, other quantitative ratios, and postvoid late images have not been evaluated separately or in conjunction with T1/2. Therefore, prospective and standardized examinations are required for further comparisons.

In this study, we compared the MRU-based RTT value with the T1/2 parameter in terms of obstruction prediction by scintigraphy. The combined use of findings and parameters will also increase the predictability of surgical intervention in children [37]. Sussman et al. reported that a T1/2 value shorter than 5 min is associated with a condition that does not cause obstruction, while a T1/2 value longer than 75 min may be compatible with obstruction that predicts pyeloplasty [38]. The combined use of findings and parameters will also increase the predictability of surgical intervention [37]. In addition, gravity-assisted delayed imaging is presented as a better predictor of surgery than T1/2 in a recent study investigating children [37].

Although antenatal hydronephrosis has been the most frequently detected abnormality, encountered in about 0.5–1% of all pregnancies, spontaneous resolution is seen in more than half of the cases within the first year of life [39]. Coplen et al. reported that antenatal pelvis AP diameter higher than 15 mm would discriminate obstruction with sensitivity and specificity of 73% and 82%, respectively [40]. The given threshold requires US examination within the first 3–7 days of postnatal life [41]. Additionally, in newborns with antenatal hydronephrosis, a second US examination within 4–6 weeks is recommended, even if the first US examination is normal [42]. The AP pelvis diameter value of 14.5 mm that we found in our study for the postnatal period also remains valid for the diagnosis of obstruction.

According to the Society of Fetal Urology (SFU) grading system, a commonly used classification, dilatations of grade 1 or 2 or postnatal APRDP < 20 mm are considered low risk, while children with SFU grade 3 or postnatal APRPD in the range of 20–30 mm are rated as intermediate risk, and those with SFU grade 4 hydronephrosis or postnatal APRPD > 30 mm are rated as high risk. Both of these findings, which predict high risk, independently predict a lower likelihood of spontaneous resolution [43]. APRPD and differential renal functions on diuretic renal scintigraphy independently predict the need for surgery [44]. Diuretic renograms present an obstructive pattern when SRF is lower than 40% and/or a decrease of SRF of more than 10% compared to the previous one on serial imaging [45]. Additional criteria that may require surgical intervention for patients suspected of having obstructive hydronephrosis in the newborn include an obstructed drainage pattern on curves or prolonged T1/2, and worsening function, hydronephrosis, or drainage over time [46].

The thresholds for T1/2 values in symptomatic children have been established as 0–10 min for good drainage, 10–20 min for an indeterminate range, and > 20 min for suggesting obstruction [47]. Wong et al. reported that > 50% residual activity after 5-min gravity-assisted drainage with T1/2 > 20 min had a sensitivity and specificity of 88% and 74% for predicting obstruction in renal units [48]. In a recent study investigating the prediction of pyeloplasty, modified T1/2 criteria were used, alone or in combination with a delayed drainage image referred to as global washout (GWO). They concluded that T1/2 > 75 min or GWO < 50% are indicators of severity of obstruction and necessity of pyeloplasty, whereas a T1/2 of < 5 min or GWO > 90% could exclude the need for surgery [38]. Additionally, in terms of obstruction prediction, drainage curves exhibiting flat or rising curves may indicate high-grade obstruction [46]. Furthermore, a recent study conducted on SFU grade 3–4 antenatal hydronephrosis concluded that T1/2 > 50 min is predictive for pyeloplasty due to worsening drainage parameters on serial scintigraphies [38].

In a recent study conducted on the necessity of evaluating the parameters together and the inadequacy of each quantitative and qualitative evaluation related to MAG-3 scintigraphy, it was revealed that the MAG-SOS system (utilizing the SRF, modified T1/2 value, and renogram curve patterns) is considered an independent determinant of the indication for pyeloplasty, rather than evaluating the parameters individually (HR 0.03, p < 0.001) [49]. A recent study revealed that when using T1/2 on MAG3 of > 20 min as the gold standard reference for presenting obstruction, an RTT value higher than 6 min (360 s) on fMRU was found to be 94.1% specific and 61.90% sensitive for UPJ obstruction [35]. On the basis of RTT, drainage can be classified as normal (RTT 4 min), equivocal (4 < RTT 8 min), or obstructed (RTT > 8 min) [14]. However, in the presence of a dilated renal pelvis, the RTT may be prolonged because the contrast medium must fill the renal pelvis before passing through the UPJ [2]. In our study, we demonstrated the value of pelvic AP diameter in the diagnosis of obstruction and its correlation with T1/2 and RTT. However, it should be noted that a dilated pelvis is not only present in UPJ stenosis but can also be accompanied by megaureter, and therefore, morphological evaluation of the entire system with ultrasound is quite important. This prolongation should be kept in mind, particularly in the presence of bladder filling, megaureter, or postoperative sequelae of pelviectasis, and should be evaluated in conjunction with the findings.

There is a distinct lack of studies comparing scintigraphy with parameters such as mean transit time and CTT. Due to the lack of comparative publications evaluating these metrics and the lack of sufficient data on RTT values, we chose to focus on RTT in our preliminary study for the prediction of obstruction. Conducting further comparative studies that include quantitative parameters is crucial for advancing our understanding. Furthermore, emerging data on the SRF parameter have shown promising correlation with scintigraphy. The location and severity of obstruction are complex and diverse, further highlighting the need for comprehensive studies that account for these differences and effectively categorize them.

Our study has some limitations. First, bladder catheterization was not performed immediately before the MRU. This may have resulted in reduced excretion and could mimic an obstruction. In addition, because the MRU and scintigraphy examinations were evaluated within the scope of a retrospective study, the examinations were not performed simultaneously. Furthermore, we did not precisely categorize the patient group according to the degree and level of obstruction and dilatation. In addition, the drainage curves and other quantitative ratios have not been evaluated separately or in conjunction. Therefore, prospective and standardized examinations are required for further comparisons. Multiple subdivisions require a large sample size. To significantly enrich the literature, future research should include several subgroups along with preoperative and postoperative comparisons, as well as the analysis of quantitative parameters along with scintigraphy. This would provide a more comprehensive understanding of the outcomes.

## Conclusion

There is a significant positive correlation between SRF values obtained via MRU and MAG3 scintigraphy. RTT values obtained from MRU offer valuable diagnostic accuracy for prediction of urinary obstruction when compared with T1/2 obtained from MAG3 scintigraphy. Thus, dynamic contrast enhanced MRU-based quantitative results are helpful in determining the functional status of kidneys.

## Supplementary Information

Below is the link to the electronic supplementary material.

Graphical abstract (PPTX 179 KB)

## Data Availability

Data generated or analyzed during the current study are available from the corresponding author upon reasonable request
