# Supplementary material for: Prediction of split renal function and obstruction with magnetic resonance urography in comparison with dynamic renal scintigraphy
Source: Pediatr Nephrol. 2026 Feb 17;41(7):2043–53. doi: 10.1007/s00467-026-07211-y (PMC13197300; doi:10.1007/s00467-026-07211-y)
Supplement: Supplementary file 1 — Graphical abstract (PPTX 179 KB) [file 467_2026_7211_MOESM1_ESM.pptx]

## Slide 1
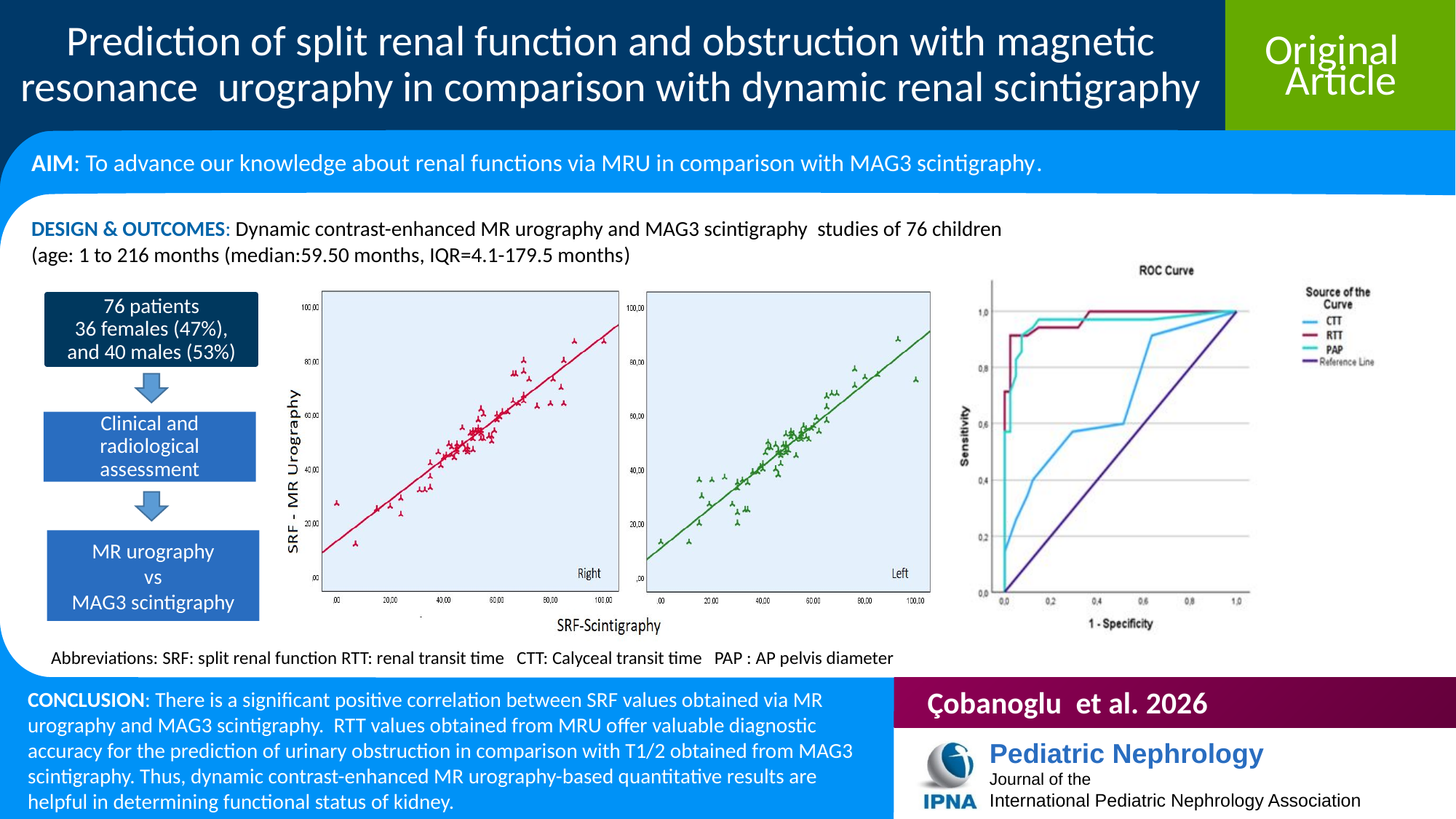

Prediction of split renal function and obstruction with magnetic resonance urography in comparison with dynamic renal scintigraphy
AIM: To advance our knowledge about renal functions via MRU in comparison with MAG3 scintigraphy.
DESIGN & OUTCOMES: Dynamic contrast-enhanced MR urography and MAG3 scintigraphy studies of 76 children
(age: 1 to 216 months (median:59.50 months, IQR=4.1-179.5 months)
76 patients
36 females (47%), and 40 males (53%)
Clinical and radiological
assessment
MR urography
vs
MAG3 scintigraphy
Abbreviations: SRF: split renal function RTT: renal transit time CTT: Calyceal transit time PAP : AP pelvis diameter
Çobanoglu et al. 2026
CONCLUSION: There is a significant positive correlation between SRF values obtained via MR urography and MAG3 scintigraphy. RTT values obtained from MRU offer valuable diagnostic accuracy for the prediction of urinary obstruction in comparison with T1/2 obtained from MAG3 scintigraphy. Thus, dynamic contrast-enhanced MR urography-based quantitative results are helpful in determining functional status of kidney.
